# Domain wall of a ferromagnet on a three-dimensional topological insulator

**DOI:** 10.1038/srep13638

**Published:** 2015-09-01

**Authors:** Ryohei Wakatsuki, Motohiko Ezawa, Naoto Nagaosa

**Affiliations:** 1 Department of Applied Physics, University of Tokyo, 7-3-1, Hongo, Bunkyo-ku, Tokyo 113-8656, Japan; 2 RIKEN Center for Emergent Matter Science (CEMS), Wako, Saitama 351-0198, Japan

## Abstract

Topological insulators (TIs) show rich phenomena and functions which can never be realized in ordinary insulators. Most of them come from the peculiar surface or edge states. Especially, the quantized anomalous Hall effect (QAHE) without an external magnetic field is realized in the two-dimensional ferromagnet on a three-dimensional TI which supports the dissipationless edge current. Here we demonstrate theoretically that the domain wall of this ferromagnet, which carries edge current, is charged and can be controlled by the external electric field. The chirality and relative stability of the Neel wall and Bloch wall depend on the position of the Fermi energy as well as the form of the coupling between the magnetic moments and orbital of the host TI. These findings will pave a path to utilize the magnets on TI for the spintronics applications.

The dissipationless topological currents (TIs) are the issue of current great interests. TIs and superconductors are the two representative materials which support the dissipationless currents on their surface^1^,^2^. These materials are characterized by the gapped bulk states and gapless surface or edge states due to bulk—edge or bulk—surface correspondence. The surface Weyl states of a three-dimensional (3D) TI offer an arena for various novel physical properties due to its momentum—spin locking, as described by the two-dimensional (2D) Hamiltonian,


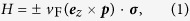


where ***e***_*z*_ is the normal unit vector to the surface, ***σ*** = (*σ*_*x*_, *σ*_*y*_, *σ*_*z*_) are the Pauli matrices, and ***p*** is the 2D momentum. The sign ± differs for the top and bottom surfaces.

This surface state shows various unique properties when magnetic moments are coupled to it. For example, the effect of the doped magnetic moments on the transport properties has been studied theoretically^3^. Another remarkable phenomena is the quantized anomalous Hall effect (QAHE), where the Hall conductance *σ*_*xy*_ is quantized with the vanishing longitudinal conductance without the external magnetic field^4^,^5^,^6^,^7^,^8^. When the exchange coupling to the magnetization is introduced, the Hamiltonian reads


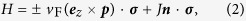


where ***J*** is the exchange energy, and ***n*** is the direction of the magnetization. When the magnetization is normal to the surface, i.e., 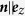, the the mass gap opens in the surface state and half-quantized Hall conductance 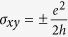, i.e., the quantized anomalous Hall effect (QAHE) is realized, when the Fermi energy is tuned within in this mass gap. Note that the observed Hall conductance is the sum of the upper and bottom surfaces and hence 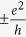.

The dynamics of the magnetization on 3D TI has been also studied theoretically based on the 2D Weyl Hamiltonian^9^,^10^,^11^,^12^,^13^,^14^,^15^,^16^. Experimentally, the gap opening in the surface states of a 3D TI Bi_2_Se_3_ due to the doping of magnetic ions has been observed by angle-resolved photoemission spectroscopy (ARPES)^17^. Also the QAHE has been recently observed in Bi_2_Te_3_ with Cr doping^18^,^19^,^20^,^21^,^22^,^23^,^24^,^25^. When the magnetization is along the *z* direction both for the top and bottom surfaces, the edge channel goes along the side surface. The edge channel appears also along the domain wall which separates the two domains of 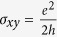 and 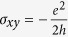.

In the field of spintronics, the magnetic domain walls play important roles as the information carriers and their manipulation is a keen issue. Especially, the racetrack memory using the current-driven motion of the domain wall is proposed^26^. Recently, the vital role of the spin—orbit interaction (SOI) in the domain wall motion has been revealed^27^. The spin-to-charge conversion by the SOI is also a hot topic in spintronics^28^. Therefore, it is an important issue to examine theoretically the domain walls in the ferromagnet on a TI from the viewpoint of the spintronics, since the momentum—spin locking at the surface state of the TI corresponds to the strong-coupling limit of the SOI.

There are some subtle issues in the Hamiltonian Eq. (2): (i) One needs to introduce the energy cut off to avoid the ultra-violet divergence, which is naturally given by the band gap of the 3D bulk states; namely, the surface states merge into the bulk conduction and valence bands. However, when the in-plane components of the magnetization *n*_*x*_, *n*_*y*_ are finite, the 2D momentum ***p*** shifts, and the surface states near the merging points are changed, which contribute to the energy but can not be properly described by Eq. (2). (ii) The exchange coupling to the magnetization in Eq. (2) needs to be re-examined. The Cr atoms replaces Bi atoms, and can have the exchange coupling to the *p*-orbitals of both Bi and Te, but with different weight. This changes the effective Hamiltonian for the surface state. (iii) The dependence on the depth of the magnetic layer, and the relation between the top and bottom surfaces are of interest as well, which is accessed only by the 3D model with finite thickness.

In this paper, we investigate the stability and charging effects of a domain wall on the surface of the 3D TI based on the 3D tight-binding model. We carry out a numerical study based on the 3D tight-binding model^29^,^30^,^31^. We also perform an analytical study based on the effective 2D surface Hamiltonian which we derive from the 3D model. The exchange coupling is found to be anisotropic due to the orbital dependence, as we have mentioned. Figure 1 shows the schematic structure of the domain wall on a TI. The angle *ϕ* determines the structure of the domain wall, i.e., Neel or Bloch wall and its chirality. It is found that the most stable domain wall structure depends on the position of the Fermi energy, i.e., one can control the domain structure by gating. Another important result is that the domain wall is charged due to the two effects: One originates in the zero-energy edge state along the domain wall and the other in the charging effect of the magnetic texture. It will offer a way to manipulate the domain wall by electric field.

## Results

### Model Hamiltonian

We start with the following minimum model for 3D TIs^29^,^30^,


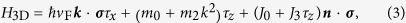


where *v*_*F*_ is the Fermi velocity, *m*_0_ and *m*_2_ are the mass parameters. For the numerical calculation, we use the corresponding lattice model^29^,^30^,^31^,


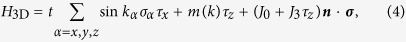


where *t* is the transfer integral, ***σ*** = (*σ*_*x*_, *σ*_*y*_, *σ*_*z*_), and ***τ*** = (*τ*_*x*_, *τ*_*y*_, *τ*_*z*_) are the Pauli matrices for the spin and pseudospin degrees of freedom, and


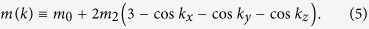


The pseudospin represents the *p*-orbitals of the Bi and Te. We have introduced an orbital-dependent exchange interaction, i.e., the *τ*_*z*_ = 1 orbital is coupled with (*J*_0_ + *J*_3_)***n*** · ***σ***, while the *τ*_*z*_ = −1 orbital with (*J*_0_ − *J*_3_)***n*** · ***σ***. When *J*_3_ = ±*J*_0_, the exchange interactions exist only at one orbital, while equally coupled when *J*_3_ = 0. In the case of Cr doped (Bi,Sb)_2_Te_3_, the magnetization is induced by the substitution of the (Bi,Sb) atoms by the Cr atoms, which is coupled mostly to the Te atoms. Hence it is expected that *J*_3_ ~ *J*_0_^32^. Therefore, we consider the two limiting cases of *J*_3_ = 0 and *J*_3_ = *J*_0_. The case *J*_3_ = 0 is useful since it provides us with a clear physical picture from the analytical point of view.

The system without the magnetism is known^29^,^30^,^31^ to be a strong TI for −12 < *m*_0/_*m*_2_ < −8 and −4 < *m*_0_/*m*_2_ < 0, a weak TI for −8 < *m*_0_/*m*_2_ < −4, and the trivial insulator for *m*_0_/*m*_2_ < −12 and 0 < *m*_0_/*m*_2_. The strong TI phase is the most intriguing, and hence we choose *m*_0_ = −0.8, *m*_2_ = 0.4 and *t* = 1 for numerical calculations and for illustration throughout the paper. The bulk gap is given by 2*m*_0_. Note that even the 4 × 4 tight-binding Hamiltonian Eq. (4) is an effective one around the top of the valence band and the bottom of the conduction band. In actual materials, there are many other bands which contribute to the higher energy and short wavelength physics. Therefore, we regard the “lattice constant” (which is put to be unity) as the coarse grained one.

We are interested in the low-energy physics on the surface of the above TI. We consider a slab geometry with finite thickness along the *z* direction. Then, the 2D Weyl fermions appear both on the top and bottom surfaces. This can be seen from the 2D low-energy Hamiltonian by projecting the 3D continuum Hamiltonian (3) onto the space spanned by the surface states. The result is


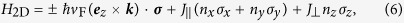


with the parameters 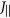 and 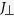, which are related with *J*_0_ and *J*_3_ in Eq. (3) and (4). It can be derived as follows.

At the Γ point, we obtain the surface states by solving the eigenequation (3) without the exchange terms by setting *k*_*x*_ = *k*_*y*_ = 0 and 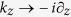 for the semi-infinite system. The top and bottom surface states are represented as


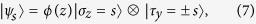


where ± in the *τ* part corresponds to the top and bottom surface respectively, and *s* = ±1 represents the spin eigenvalue. Therefore, we find


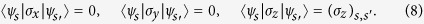


It follows that 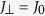, namely the exchange term in the 3D bulk Hamiltonian is projected into the Ising interaction in the 2D surface Hamiltonian. For the orbital-dependent exchange term, the components are


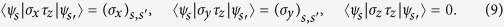


It follows that 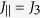, namely the orbital-dependent exchange term in the 3D bulk Hamiltonian is projected into the in-plane exchange term in the 2D surface Hamiltonian.

Our important observation is that the exchange interaction on the 2D surface is anisotropic even if that in the 3D bulk is isotropic. The perpendicular exchange interaction is induced by the *J*_0_-term while the in-plane exchange interaction is induced by the *J*_3_-term.

In what follows we carry out an analysis of the surface states of the TI numerically based on the 3D Hamiltonian Eq. (4) and analytically based on the 2D Hamiltonian Eq. (6). The momentum *k*_*y*_ is a good quantum number since the surface are assumed to be uniform in the *y* direction. We numerically diagonalize the system with 128 sites along the *x* direction and 8 sites along the *z* direction for each *k*_*y*_. We take 200 points for *k*_*y*_. We set two domain walls to apply the periodic boundary condition for *x* direction, and we illustrate figures for one of the domain walls throughout the paper.

### Magnetic domain wall

We consider a magnetic domain wall between the two degenerate ground states, ***n*** = ±(0, 0, 1) lying along the *y* axis on the surface of the TI,


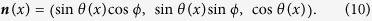


with 
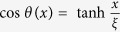
. The angle *ϕ* represents the type of magnetic domain wall. Especially, *ϕ* = 0, *π* represent the Neel walls, while *ϕ* = *π*/2, 3*π*/2 the Bloch walls,


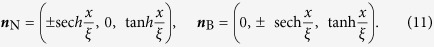


We call *ϕ* = 0 (*ϕ* = *π*) as Neel 1 (Neel 2), and *ϕ* = *π*/2, 3*π*/2 as Bloch. These two types of Bloch walls are related by the mirror symmetry operation with respect to the *zx* plane.

The domain wall width *ξ* should be optimized as a variational parameter in Eq. (10). It is found that the energy is decreased as *ξ* is decreased down to *ξ* = 2.0. See Supplementary Information S1. Therefore, the width of the domain wall is typically the lattice constant in this model. The reason is basically that the kinetic energy in the 2D effective Hamiltonian is solely given by the SOI and hence there is no length scale due to the SOI other than the lattice constant. The detailed discussion is given in Supplementary Information S2. However, as mentioned above, the lattice constant of the present tight-binding Hamiltonian is that of the coarse grained model, and hence the distinction between Neel and Bloch walls still makes sense. Also the width depends on the additional single-ion magnetic anisotropy term 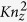 which exists in the real material but not included in the present model. We have numerically confirmed that the qualitative features of the results do not depend on *ξ*, and hence we have shown the results for *ξ* = 4.0 for illustrative purpose in order to clearly show the difference between the Neel and Bloch walls. See S1 in Supplementary Information.

### Edge modes

**Figure 2:**
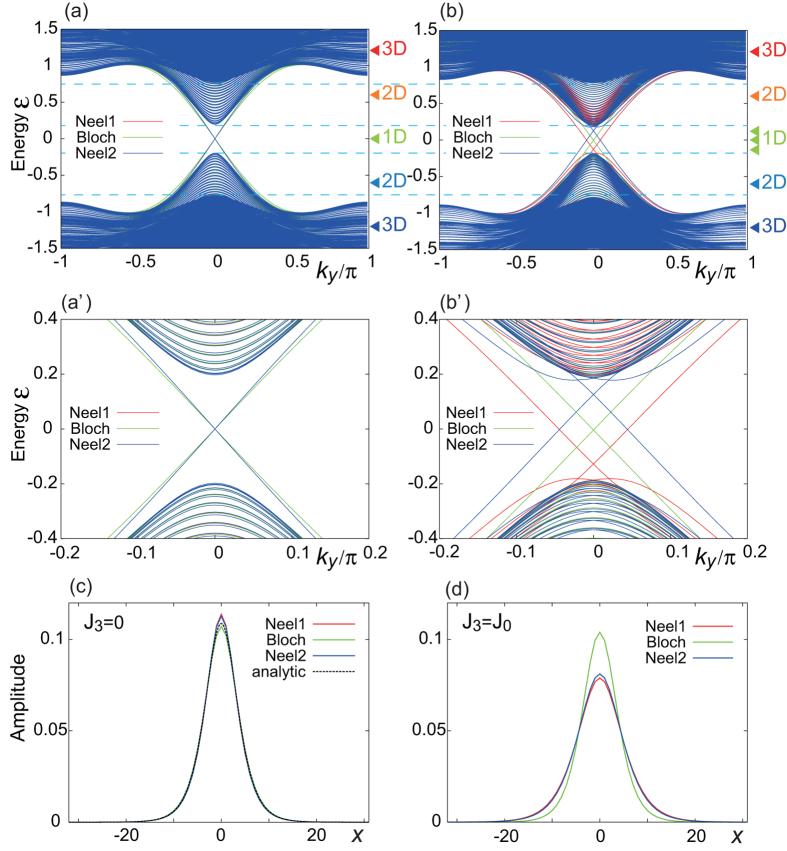
The energy dispersions of the bulk states and edge modes for (a), (a’) *J*_3_ = 0 and (b), (b’) *J*_3_ = *J*_0_. The edge mode appears inside the bulk band gap. The dispersion is almost independent of *ϕ*, i.e., the type of domain wall, for *J*_3_ = 0 (**a**), (**a’**), while it is sensitive when *J*_3_ = *J*_0_ (**b**), (**b’**). Probability distribution of the zero-energy wave function for *k*_*y*_ = 0 for (**c**) *J*_3_ = 0, (**d**) *J*_3_ = *J*_0_. The dotted curve in (**c**) is given by Jackiw—Rebbi solution Eq. (12) in the text.

A magnetic domain wall separates the two domains with up and down spins, i.e., the regions of 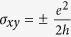. Therefore, the difference of *σ*_*xy*_ is 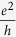 and hence one chiral edge channel is expected to appear along the domain wall. We show the energy dispersion and the probability distribution of the edge channel wave function along the *x* direction obtained numerically for *J*_3_ = 0 in Fig. 2(a,a’), and *J*_3_ = *J*_0_ in Fig. 2(b,b’), respectively.

There are three energy scales in the band structure as shown in Fig. 2(a,b). One is the 3D bulk band structure which exists for 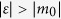. The second is the 2D surface band structure which exists for 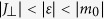. The last is the 1D edge states along the domain wall which exists for 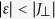.

When *J*_3_ = 0, the dispersion and wave function of the edge modes are almost independent of *ϕ* as shown in Fig. 2(a,a’). This is consistent with Eq. (6) with 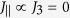. Since the coupling is Ising-like, there is no *ϕ* dependence for the surface states. We have determined numerically the probability distribution of the wave function at *k*_*y*_ = 0, which we show in Fig. 2(c,d).

We present a clear physical picture for the zero-energy edge mode for 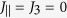. The wave function is analytically given by the Jackiw—Rebbi solution^33^,


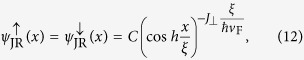


with a normalization constant *C*. It can be obtained by solving the differential equation given from Eq. (6)


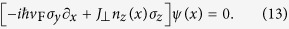


Indeed, it well explains the numerical data in Fig. 2(c). The half width of the wave function is the same order of the domain wall width *ξ*.

On the other hand, the edge modes depend on *ϕ* when *J*_3_ = *J*_0_ as shown in Fig. 2(b,b’). This is again consistent with Eq. (6) with 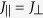. The energy dispersion of the edge mode is well described by


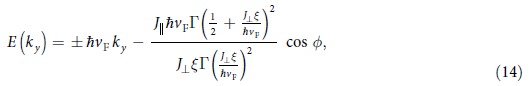


as we derive by the first-order perturbation in Supplementary Information S3. Note that the spatial extent of the wave function is affected by the energy separation between the in-gap state and the edge of the bulk density states, and hence depends on *ϕ* in this case.

### Domain wall energy

**Figure 3:**
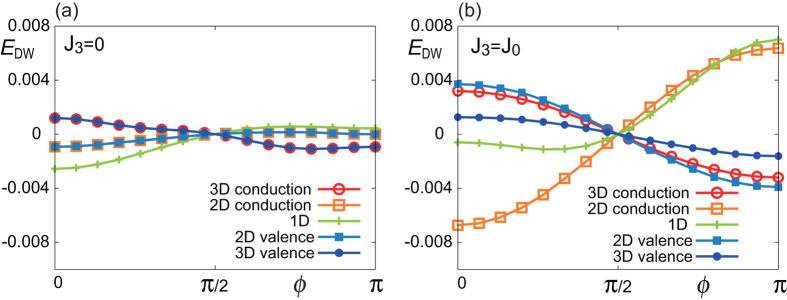
The energy of the domain wall *E*_DW_ as a function of *ϕ* for (a) *J*_3_ = 0, (b) *J*_3_ = *J*_0_. When *J*_3_ = 0, the lowest energy domain wall structure is at *ϕ* = 0 for *μ* in the 2D valence/conduction bands or inside the gap. It turns into *ϕ* = *π* when *μ* is in the 3D valence/conduction bands. When *J*_3_ = *J*_0_, on the other hand, *ϕ* = 0 is most stable when *μ* is in the 2D conduction band, and nearly Bloch wall 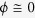 is stable for *μ* inside the gap. *ϕ* = *π* is the most stable for other cases. This behavior can be understood by considering the DM derived from the 2D surface states.

We show in Fig. 3 the *ϕ*-dependence of the domain wall energy *E*_DW_ measured from the value at *ϕ* = *π*/2 (Bloch wall) for several values of the chemical potential *μ* when the magnetic layer is at the top surface. (The absolute value of the domain wall energy compared with the uniform magnetization is a more subtle quantity, which depends also on the magnetic anisotropy term 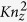, and therefore we do not address it in this paper.) The domain wall energy is the same for *ϕ* and 2*π* − *ϕ* due to the mirror symmetry with respect to *zx* plane as 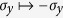, 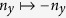. Therefore, it is enough to show the results for 0 ≤ *ϕ* ≤ *π*. *E*_DW_(*ϕ*) behaves quite differently between the cases of *J*_3_ = 0 and *J*_3_ = *J*_0_. (In Supplementary Information S4, Fig. S4 illustrates *E*_DW_ for various values of *J*_3_.) When *J*_3_ = 0, the Neel wall with *ϕ* = 0 is the most stable for the chemical potential *μ* in the 2D valence/conduction bands or inside the gap. When *μ* is in the 3D bands, the Neel wall with *ϕ* = *π* becomes the most stable. In this case, the system possesses the particle—hole symmetry as shown in Supplementary Information S5. As a result, the energy is symmetric between 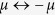, which is also verified by our numerical calculations.

On the other hand, when *J*_3_ ≠ 0, the particle—hole symmetry is lost. For *J*_3_ = *J*_0_ in Fig. 3(b), the minimum energy configuration changes from *ϕ* = *π* (Neel 2) for large positive *μ* > 0 (in the 3D conduction band), turns to *ϕ* = 0 (Neel1) for *μ* in the 2D conduction band, approaches to *ϕ* = *π*/2 (Bloch) for *μ* within the gap, and eventually to *ϕ* = *π* (Neel 2) for *μ* < 0. This means that one can control the angle *ϕ* of the domain wall by the gate voltage, which changes the chemical potential. This is one of our main results in the present paper. This change of the stable magnetic structure is understood analytically in terms of the effective Dzyaloshinskii—Moriya (DM) interaction induced from the TI surface state as discussed below.

When the chemical potential is in the 2D surface band 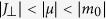, the stability of a magnetic domain wall can be understood in terms of the effective surface DM interaction due to the Weyl surface states. In order to derive the effective Hamiltonian for the magnets, we integrate out the fermion degrees of freedom, namely, calculate the following effective action


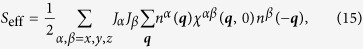


where 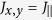, 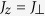, and *χ* is the spin susceptibility,


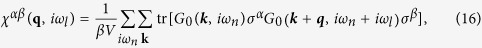


and *G*_0_ is the Green’s function for the Weyl Hamiltonian (6) without the exchange terms. We obtain


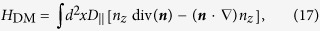


with


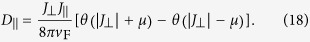


The detailed derivation is shown in Supplementary Information S2. It is zero within the band gap of the 2D surface state. The sign of the DM interaction is positive for 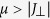 and negative for 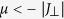. Namely, we can control the sign of the DM interaction by changing the chemical potential by the gate voltage. It is noted that the sign change of the DM interaction stems from the helicity difference of the momentum—spin locking on the conduction and valence bands. We evaluate the domain wall energy change due to the DM interaction. Substituting the domain wall texture (10) into Eq. (17), we obtain


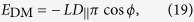


with the length of the domain wall *L*. It takes the minimum energy for the Neel domain wall with *ϕ* = 0 (*π*) for 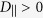 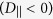.

Finally, we briefly note the general case 0 < *J*_3_ < *J*_0_. When *J*_3_/*J*_0_ increases from zero, the exchange interaction on the surface changes from the Ising-like anisotropic form to the Heisenberg-like isotropic form. Therefore, the energy difference among the various domain walls continuously increases. The numerical results are shown in Supplementary Information S4.

### Electron density distribution

**Figure 4:**
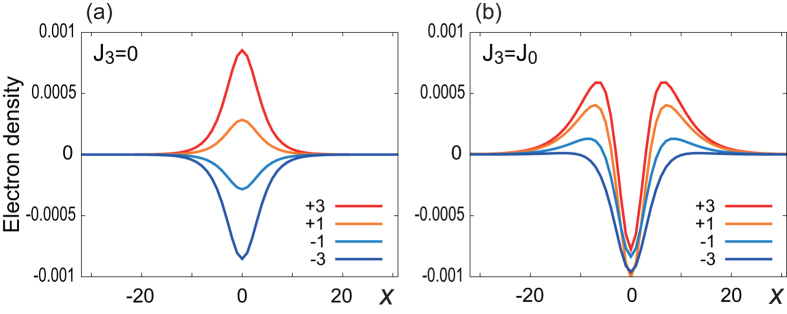
Electron density distribution of the optimized domain wall structure for various chemical potential for (a) *J*_3_ = 0 and (b) *J*_3_ = *J*_0_. Electrons (holes) are localized at the zero-energy states due to the magnetic domain wall for *μ* > 0 (*μ* < 0). The numbers ±1, ±3 indicate the electron number measured from the half-filling. The horizontal axis is the *x* coordinate.

We demonstrate in Fig. 4 the electron density distribution of the upper half layers for the minimum-energy domain wall configuration numerically calculated via the expression


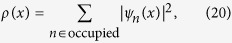


where {*ψ*_*n*_} are the eigenfunctions of the 3D Hamiltonian with the band index *n*. (For the electron density distributions corresponding to general domain wall configurations, see Supplementary Information S6) When *J*_3_ = 0 (Fig. 4(a), the density distribution is uniform for *μ* = 0, while it is localized at the domain wall for *μ* ≠ 0 inside the bulk band gap. The density distribution is inverted between 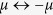.

We may explain the electron accumulation analytically as follows. For *J*_3_ = 0 the edge state is well described by the Jackiw—Rebbi mode Eq. (12). It gives the edge channel wave function at zero energy for electrons or holes. When the chemical potential *μ* is shifted, the electrons or holes accumulate into the edge states for *μ* within the 2D surface band gap. Hence, by considering the density of states for the edge channel, the electron density is given by


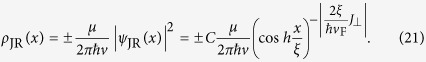


On the other hand, when *J*_3_ = *J*_0_, there are two peaks in the density distribution of the Neel domain wall as found in Fig. 4(b). To understand this behavior, we recall that the electron accumulation due to the spin texture has previously been shown to be^34^


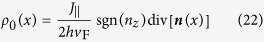


for a smooth magnetic texture *n*_*z*_(≠0) which remains almost constant for all over the sample. Following Ref. 26, this relation can be derived by considering the Chern—Simons action


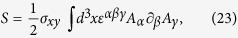


and the gauge field is


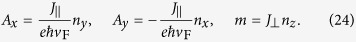


By using the relation 
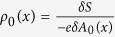
, Eq. (22) is be obtained.

The total accumulation must consist of the zero-energy edge contribution *ρ*_JR_(*x*) and the background contribution *ρ*_0_(*x*), *ρ*(*x*) = *ρ*_JR_(*x*) + *ρ*_0_(*x*). The Neel-type magnetic configurations contributes to the electron accumulation *ρ*_0_(*x*), but that there is no such an accumulation in the Bloch-type magnetic configurations because *n*_*x*_ = 0. Thus, *ρ*_0_(*x*) may be the difference of the electron accumulation between the Neel and Bloch domain walls. In our case, *ρ*_0_ for the Neel wall is


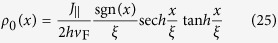


with the use of 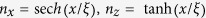 in Eq. (22) for a Neel domain wall.

**Figure 5:**
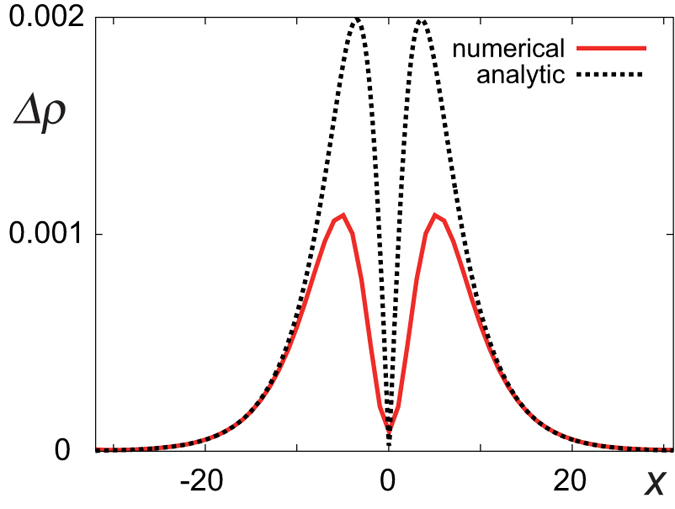
Difference of the electron density distribution Δ*ρ*(*x*) (red curve) between the Neel and the Bloch domain wall with equal chemical potential *μ*. The horizontal axis is the *x* coordinate. It is well explained by the formula Eq. (25) semi-quantitatively as shown by a black dotted curve. Especially, the dotted curve fits perfectly at tails, where the formula Eq. (25) is expected to be accurate.

To confirm this scenario, we plot the difference in the charge density Δ*ρ*(*x*) between the Neel and Bloch walls with the equal chemical potential in Fig. 5. The formula Eq. (25) captures the key structure of the numerical data as in Fig. 5. Therefore, the peculiar double peak structure in Fig. 4(b) stems from the combination of the chiral edge channel and the spatial variation of the spin texture. The amplitude can be enhanced or reduced, depending on the domain wall type and the filling of the edge channel.

Finally, we note that we can estimate the charging energy with the obtained electron distributions, and conclude that the charging energy is negligible compared with the band energy. The detailed discussion is shown in Supplementary Information S7.

## Discussions

The origin of the ferromagnetism in doped TI is an important issue. A first-principles calculation on Mn-doped Bi_2_Te_3_^30^ indicates that the Hamiltonian Eq. (2) is a good effective model for the surface states. The gap depends strongly on the direction of the magnetization ***M***; it is ~16 meV when ***M*** is perpendicular to the surface, while the shift in the in-plane momentum 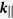 occurs when ***M*** is parallel to the surface. From the comparison between the gap in the former case and the energy shift at 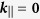 in the latter case, it is concluded that 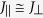 in Eq. (2), i.e., 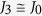. Physically, the Cr and Mn atoms are replacing Bi, and probably the coupling to the neighboring Te *p*-orbitals are stronger than to those of Bi atoms, which results in this orbital dependent exchange interaction. Experimentally, the gating can tune the chemical potential *μ* and it has been argued from the dependence of ferromagnetic *T*_c_ on *μ* that the coupling to the surface Weyl fermions is the origin of the ferromagnetism^35^. Therefore, the model Eq. (2) is appropriate also from this viewpoint.

However, in real materials, the magnetic ions are not selectively doped on the surface but are distributed in the whole sample. Therefore, it is expected that the magnetization behaves uniformly along the *z* direction (perpendicular to the surface) for the thin film samples with the thickness of the order of 8 nm^20^. The bulk mechanism of ferromagnetism in doped TI is studied theoretically also^6^,^36^. In Supplementary Information S8, we study the dependence on the depth of the magnetic layer. When the magnetization on the top and bottom surfaces are the same, the energies of *ϕ* = 0 domain wall (Neel 1) and *ϕ* = *π* domain wall (Neel 2) are degenerate because of the mirror symmetry with respect to the plane separating the upper and lower halves of the film. This argument, however, assumes the equivalence between the top and bottom surfaces, which is not satisfied in general experimental setups. Actually, it is observed that the Weyl points on top and bottom surfaces are different in energy typically of the order of 50 meV^37^, and this symmetry is broken. Therefore, we expect that the type of the domain wall can be manipulated by gating.

As for the existence of the domain walls, they are naturally introduced in the hysteresis loop in the magnetic field - magnetization curves. Actually the longitudinal resistance *R*_*xx*_ is found to have the peak 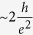 at the ends of the hysteresis loop, which is likely due to the chiral edge channel associated with the domain wall^20^. An interesting possibility is the formation of skyrmions, which corresponds to the circular closed loop of a domain wall. It is well known the charge doping into *v* = 1 quantum Hall ferromagnet results in the formation of skyrmions^38^. It remains an open issue if the skyrmions can appear in the quantized anomalous Hall system on 3D TI.

## Methods

We have used the 3D Hamiltonian Eq. (4) for the numerical calculations. We assume the periodic boundary condition for the *x* and *y* directions, and the open boundary condition for the *z* direction. We put non-uniform magnetic moments for the *x* direction. Therefore, *k*_*y*_ is a good quantum number. By summing up eigenenergies and amplitudes of eigenfunctions below a certain particle number, we obtain the total energy and the electron density distribution. We set the zero of the energy for that of the Bloch wall, and the zero of the density for that of the half-filling case. In Fig. 5(c), we compared the density of a Neel wall and a Bloch wall with the same chemical potential. We set *t* = 1, *m*_0_ = −0.8, *m*_2_ = 0.4, *J* = 0.2, *ξ* = 4 for the main text.

## Additional Information

**How to cite this article**: Wakatsuki, R. *et al.* Domain wall of a ferromagnet on a three-dimensional topological insulator. *Sci. Rep.* **5**, 13638; doi: 10.1038/srep13638 (2015).

## Supplementary Material

Supplementary Information

## Figures and Tables

**Figure 1:**
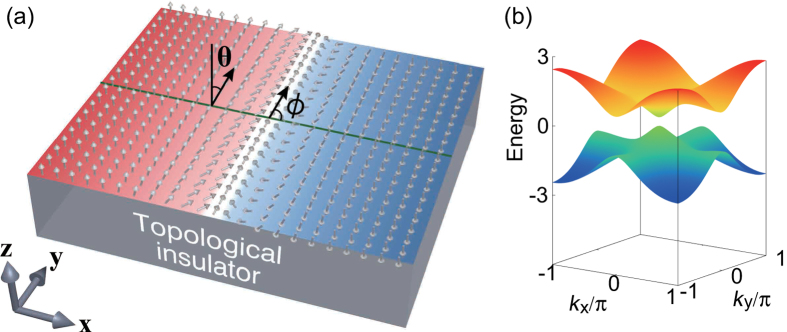
(**a**) Illustration of the domain wall in the ferromagnet on a TI. Along the domain wall, the gapless chiral edge channel appears (white stripe region). The angle *ϕ* specifies the type of the domain wall, i.e., *ϕ* = 0, *π* corresponds to Neel wall while *ϕ* = *π*/2, 3*π*/2 to Bloch wall. (**b**) The surface band structure with homogeneous ferromagnetic calculated from the 3D tight-binding model. The vertical axis is the energy in unit of *t*.
