# Supplementary material for: Domain wall of a ferromagnet on a three-dimensional topological insulator
Source: Sci Rep. 2015 Sep 1;5:13638. doi: 10.1038/srep13638 (PMC4555097; doi:10.1038/srep13638)
Supplement: Supplementary Information [file srep13638-s1.pdf]

Supplementary Information

Domain wall of a ferromagnet on a three-dimensional topological insulator

Ryohei Wakatsuki[1], Motohiko Ezawa[1], and Naoto Nagaosa[1,2]
[1] *Department of Applied Physics, University of Tokyo,*
7-3-1, Hongo, Bunkyo-ku, Tokyo 113-8656, Japan and
[2] *RIKEN Center for Emergent Matter Science (CEMS), Wako, Saitama 351-0198, Japan*

S1. Width dependence of the domain wall energy

Figure S1 shows the domain wall energy $E_{\rm DW}$ with the width $\xi = 2.0$ as a function of ϕ for $J_3 = 0$ ((a)) and $J_3 = J_0$ ((b)). The qualitative behavior is the same as that of Fig. 3 for $\xi = 4.0$ in the main text. Figure S2 shows $E_{\rm DW}$ as a function of ξ. It is seen that the smaller ξ is preferred for all the cases. For the illustrative purpose, we set $\xi = 4.0$ in the main text so that we can capture the difference between various domain wall structures.

S2. Effective action

We derive an effective action of the 2D Weyl Hamiltonian. The effective action is obtained by calculating the susceptibility in the one-loop approximation,

$$S_{\rm eff} = \frac{1}{2} \sum_{\alpha,\beta=x,y,z} J_\alpha J_\beta \sum_{\boldsymbol{q}} n^\alpha(\boldsymbol{q}) \chi^{\alpha\beta}(\boldsymbol{q},0) n^\beta(-\boldsymbol{q}), \tag{S1}$$

where $J_{x,y} = J_\parallel$ and $J_z = J_\perp$. For the region $qv_{\rm F} \ll |m|$, one can expand $\chi^{\alpha\beta}(\boldsymbol{q},0)$ with respect to $\boldsymbol{q}$, while for the region $qv_{\rm F} \gg |m|$, the mass m can be put to be zero for the derivation.

We derive the DM interaction and the exchange interaction by calculating the non-diagonal element of $\chi^{\alpha\beta}$ in the massive Weyl Hamiltonian. We estimate the domain wall width by calculating the diagonal element of $\chi^{\alpha\beta}$ in the massless Weyl Hamiltonian.

The DM and exchange interaction:

Firstly, we derive the DM interaction and the exchange interaction from the massive Weyl Hamiltonian. The continuum theory of the 2D Hamiltonian is described by

$$H = v_{\rm F} \left(\boldsymbol{e}_z \times \boldsymbol{k}\right) \cdot \boldsymbol{\sigma} + m\sigma_z. \tag{S2}$$

The Green's function reads

$$G_0\left(\boldsymbol{k}, i\omega_n\right) = \frac{1}{i\omega_n - \mu - H} = \frac{\left(i\omega_n - \mu\right)\mathbb{I} + v_{\rm F}\left(\boldsymbol{e}_z \times \boldsymbol{k}\right)\cdot\boldsymbol{\sigma} + m\sigma_z}{\left(i\omega_n - \mu\right)^2 - v_{\rm F}^2 \boldsymbol{k}^2 - m^2}. \tag{S3}$$

With the use of the Green's function, the susceptibility is given by

$$\chi^{\alpha\beta}(\boldsymbol{q}, i\omega_l) = \frac{1}{\beta V} \sum_{i\omega_n} \sum_{\boldsymbol{k}} \mathrm{tr}\left[G_0\left(\boldsymbol{k}, i\omega_n\right)\sigma^\alpha G_0\left(\boldsymbol{k}+\boldsymbol{q}, i\omega_n + i\omega_l\right)\sigma^\beta\right]. \tag{S4}$$

We put $i\omega_l = 0$, and take the $T = 0$ limit. We can show

$$\chi^{zx}(\boldsymbol{q},0) = -i\tilde{D}_\parallel q_x, \quad \chi^{zy}(\boldsymbol{q},0) = -i\tilde{D}_\parallel q_y, \quad \chi^{xz}(\boldsymbol{q},0) = +i\tilde{D}_\parallel q_x, \quad \chi^{yz}(\boldsymbol{q},0) = +i\tilde{D}_\parallel q_y \tag{S5}$$

with

$$\tilde{D}_\parallel = \frac{1}{4\pi v_{\rm F}}\left[\theta\left(|m| + \mu\right) - \theta\left(|m| - \mu\right)\right]. \tag{S6}$$

The susceptibility is inverted between $\mu \longleftrightarrow -\mu$. The DM interaction reads

$$H_{\rm DM} = D_\parallel \int dxdy \left[n_z\left(\frac{\partial n_x}{\partial x} + \frac{\partial n_y}{\partial y}\right) - n_x\frac{\partial n_z}{\partial x} - n_y \frac{\partial n_z}{\partial y}\right], \tag{S7}$$

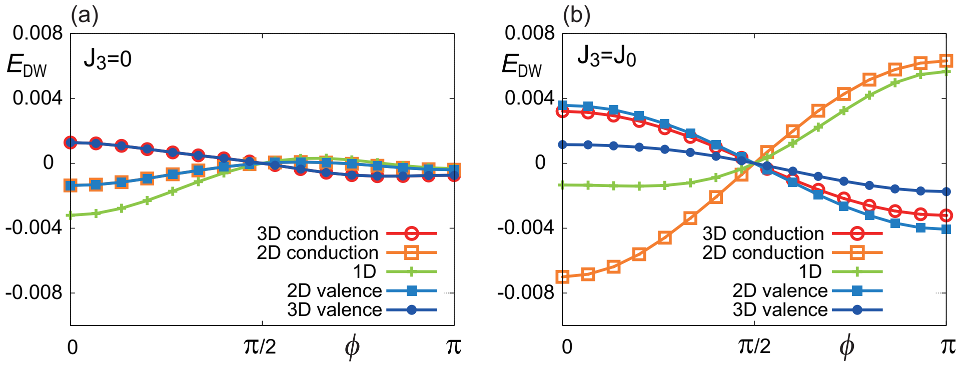

FIG. S1: Domain wall energy $E_{\rm DW}$ as a function of ϕ for $\xi = 2.0$. The qualitative behavior does not depend on ξ.

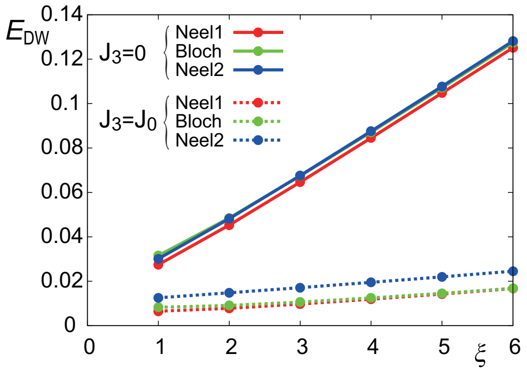

FIG. S2: Domain wall energy $E_{\rm DW}$ as a function of ξ for half-filled case. This model prefers smaller ξ.

with $D_{\parallel} = \frac{J_\perp J_\parallel}{2}\tilde{D}_{\parallel}$. This is Eq. (18) in the main text.

We calculate the diagonal terms $\chi^{\alpha\alpha}(\boldsymbol{q},0)$ up to $O(q^2)$,

$$\chi^{xx}(\boldsymbol{q},0) = -\frac{\Lambda}{3\pi^2 v_{\rm F}} + \tilde{J}q_x^2, \qquad \chi^{yy}(\boldsymbol{q},0) = -\frac{\Lambda}{3\pi^2 v_{\rm F}} + \tilde{J}q_y^2, \qquad \chi^{zz}(\boldsymbol{q},0) = -\frac{\Lambda}{\pi^2 v_{\rm F}} + \tilde{J}\left(q_x^2 + q_y^2\right), \tag{S8}$$

with

$$\tilde{J} = \frac{1}{12\pi|m|}\left[\theta\left(\mu + |m|\right) - \theta\left(\mu - |m|\right)\right]. \tag{S9}$$

They contain the ultra-violet momentum cut-off Λ, which is naturally given by the 3D band or the lattice constant. The q-independent terms yield

$$H_K = -\frac{\Lambda}{3\pi^2 v_F}\int dxdy\left[J_\parallel^2\left(n_x^2 + n_y^2\right) + 3J_\perp^2 n_z^2\right], \tag{S10}$$

which describes the easy-axis anisotropy, while the q-dependent terms yield

$$H_{\rm Ex} = \tilde{J}\int dxdy\left\{J_\parallel^2\left[\left(\frac{\partial n_x}{\partial x}\right)^2 + \left(\frac{\partial n_y}{\partial y}\right)^2\right] + J_\perp^2\left[\left(\frac{\partial n_z}{\partial x}\right)^2 + \left(\frac{\partial n_z}{\partial y}\right)^2\right]\right\}, \tag{S11}$$

which acts as the exchange interaction.

The domain wall width:

Next, we derive the effective action for $\mu = 0, m = 0$. We make the following decomposition of Eq. (S4),

$$\chi^{\alpha\beta}(\boldsymbol{q},0) = \chi^{\alpha\beta}(\boldsymbol{0},0) + \left[\chi^{\alpha\beta}(\boldsymbol{q},0) - \chi^{\alpha\beta}(\boldsymbol{0},0)\right]. \tag{S12}$$

We calculate the $I^{\alpha\beta}(\boldsymbol{q}) \equiv \left[\chi^{\alpha\beta}(\boldsymbol{q},0) - \chi^{\alpha\beta}(\boldsymbol{0},0)\right]$ term,

$$I^{zz}(\boldsymbol{q}) = \frac{2}{v_{\mathrm{F}}} \int_0^1 dx \int \frac{d^3k}{(2\pi)^2} \frac{(1-x)\,q^2}{\left[k^2 + x\,(1-x)\,q^2\right]^2} = \frac{q}{8v_{\mathrm{F}}}, \tag{S13}$$

and so on. The results for Eq. (S12) read up to $O(q^2)$ as

$$\chi^{xx}(\boldsymbol{q}) = -\frac{\Lambda}{3\pi^2 v_{\mathrm{F}}} + \frac{q_x^2}{16 v_{\mathrm{F}} q}, \quad \chi^{yy}(\boldsymbol{q}) = -\frac{\Lambda}{3\pi^2 v_{\mathrm{F}}} + \frac{q_y^2}{16 v_{\mathrm{F}} q}, \quad \chi^{zz}(\boldsymbol{q}) = -\frac{\Lambda}{\pi^2 v_{\mathrm{F}}} + \frac{q}{8 v_{\mathrm{F}}}, \tag{S14}$$

$$\chi^{xy}(\boldsymbol{q}) = \chi^{yx}(\boldsymbol{q}) = \frac{q_x q_y}{16 v_{\mathrm{F}}\, q}, \quad \chi^{zx}(\boldsymbol{q}) = \chi^{xz}(\boldsymbol{q}) = \chi^{zy}(\boldsymbol{q}) = \chi^{yz}(\boldsymbol{q}) = 0. \tag{S15}$$

The domain wall width ξ is estimated as follows. First, assume that $q \sim 1/\xi$ is much smaller than $|m|/v_F$, and use the expansion Eqs. (S15) and (S17). Then, we demand the anisotropy energy, i.e., the difference between $\chi^{zz}(0,0)$ and $\chi^{xx}(0,0) = \chi^{yy}(0,0)$, is equal to the elastic energy. However, the obtained q is much larger than $|m|/v_F$ since $|m| \gg v_F\Lambda$. Therefore, we need to look for $q \sim 1/\xi$ in the region $q \gg |m|/v_F$, i.e., using Eq. (S21). This results in

$$\xi = \frac{\pi^2}{8\Lambda} \simeq a, \tag{S16}$$

which satisfies $v_F/\xi \gg |m|$ and hence gives the self-consistent estimation.

S3. Edge channel along the domain wall

We investigate the edge channel along the domain wall,

$$n_x = \cos\phi \operatorname{sech}\frac{x}{\xi}, \quad n_y = \cos\phi \operatorname{sech}\frac{x}{\xi}, \quad n_z(x) = \tanh\frac{x}{\xi}. \tag{S17}$$

The exact solution is obtained when $J_{\|} = 0$, which is known as the Jackiw-Rebbi solution. The continuum theory of the 2D Hamiltonian in Eq. (2) is described by

$$H = v_{\mathrm{F}}\,(\boldsymbol{e}_z \times \boldsymbol{k}) \cdot \boldsymbol{\sigma} + J_\perp n_z(x) \sigma_z. \tag{S18}$$

The eigenequation for $k_y = 0, E = 0$ is

$$\begin{pmatrix} J_\perp n_z & -v_F \partial_x \\ v_F \partial_x & -J_\perp n_z \end{pmatrix} \begin{pmatrix} \psi_\uparrow(x) \\ \psi_\downarrow(x) \end{pmatrix} = 0. \tag{S19}$$

The solution which is localized around the domain wall is

$$\psi_{\uparrow,\downarrow}(x) = C \left(\cosh\frac{x}{\xi}\right)^{-J_\perp \frac{\xi}{v_{\mathrm{F}}}}, \tag{S20}$$

which is Eq. (12) in the main text, with the normalization constant

$$C^2 = \frac{\Gamma\left(\frac{1}{2} + \frac{J_\perp \xi}{v_{\mathrm{F}}}\right)}{\sqrt{\pi}\xi\Gamma\left(\frac{J_\perp \xi}{v_{\mathrm{F}}}\right)}. \tag{S21}$$

In the presence of n_x and n_y terms for small $J_{\|}$, we may estimate the energy as

$$\langle\psi| \frac{J_{\|}}{\cosh\frac{x}{\xi}} |\psi\rangle = \frac{J_{\|} v_{\mathrm{F}} \Gamma\left(\frac{1}{2} + \frac{J_\perp \xi}{v_{\mathrm{F}}}\right)^2}{J_\perp \xi \Gamma\left(\frac{J_\perp \xi}{v_{\mathrm{F}}}\right)^2}, \tag{S22}$$

which is in Eq. (14) in the main text.

Figure S3 shows numerical results on the shift of energy at $k_y = 0$ as a function of ϕ. They are well fitted by the cosine curve obtained by the first-order perturbation Eq. (S22) for $J_3/J_0 = 0.2$.

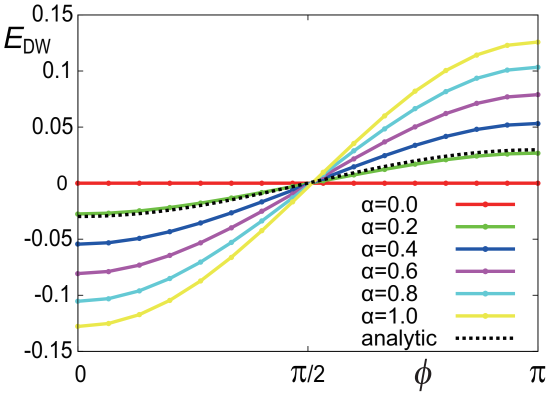

FIG. S3: Domain wall energy at $k_y = 0$ for various $\alpha \equiv J_3/J_0$. The black dotted curve is the result of the first-order perturbation for $\alpha = 0.2$.

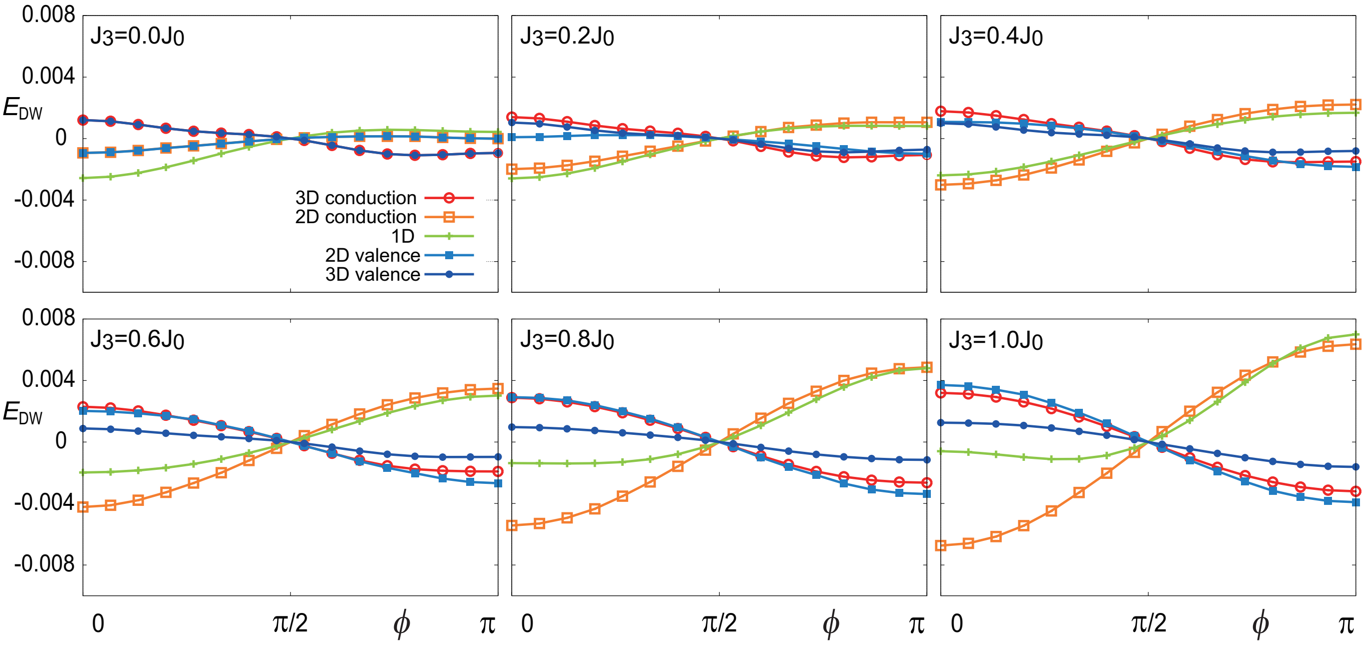

FIG. S4: Domain wall energy E_{DW} as a function of ϕ for various J_3.

S4. J_3 dependence of the domain wall energy

Figure S4 shows the set of figures corresponding to Fig. 3 in the main text with various J_3.

S5. Symmetry Analysis

Here, we summarize the symmetry properties of the models presented in the main text.

3D Hamiltonian:

The 3D tight-binding Hamiltonian in Eq. (4) is given by

$$H_{3D}\left(\boldsymbol{k}, \boldsymbol{n}, J_0, J_3, \mu\right) = t\tau_x\left(\sigma_x \sin k_x + \sigma_y \sin k_y + \sigma_z \sin k_z\right) + m\tau_z + (n_x\sigma_x + n_y\sigma_y + n_z\sigma_z)(J_0 + J_3\tau_z) - \mu, \tag{S23}$$

where μ is the chemical potential. Its complex conjugate is

$$\begin{aligned} &K H_{3D}\left(\boldsymbol{k}, \boldsymbol{n}, J_0, J_3, \mu\right) K = H_{3D}^{*}\left(\boldsymbol{k}\right) \\ &= t\tau_x\left(\sigma_x \sin k_x - \sigma_y \sin k_y + \sigma_z \sin k_z\right) + m\tau_z + (n_x\sigma_x - n_y\sigma_y + n_z\sigma_z)(J_0 + J_3\tau_z) - \mu. \end{aligned} \tag{S24}$$

We consider an operator

$$\Upsilon = \tau_x \sigma_y, \tag{S25}$$

which transforms the Hamiltonian as

$$\Upsilon H_{3D}^* \left(\boldsymbol{k}, \boldsymbol{n}, J_0, J_3, \mu\right) \Upsilon^{-1} = -H_{3D} \left(\boldsymbol{k}, \boldsymbol{n}, J_0, -J_3, -\mu\right). \tag{S26}$$

On the other hand, the particle-hole operator

$$\Xi = \tau_y \sigma_y K \tag{S27}$$

transforms the Hamiltonian as

$$\Xi H_{3D} \left(\boldsymbol{k}, \boldsymbol{n}, J_0, J_3, \mu\right) \Xi^{-1} = \tau_y \sigma_y H_{3D}^* \left(\boldsymbol{k}\right) \sigma_y \tau_y = -H_{3D} \left(-\boldsymbol{k}, \boldsymbol{n}, J_0, -J_3, -\mu\right). \tag{S28}$$

This operator is the generator of the particle-hole transformation together with $\boldsymbol{k} \to -\boldsymbol{k}$, $J_3 \to -J_3$ and $\mu \to -\mu$. The energy spectrum is symmetric (asymmetric) between the positive and the negative energy when $J_3 = 0$ ($J_3 \neq 0$).

The time-reversal operator is given by

$$\Theta = i\sigma_y K, \tag{S29}$$

which transforms the Hamiltonian as

$$\Theta H_{3D} \left(\boldsymbol{k}, \boldsymbol{n}, J_0, J_3, \mu\right) \Theta^{-1} = \sigma_y H_{3D}^* \left(\boldsymbol{k}\right) \sigma_y = H_{3D} \left(-\boldsymbol{k}, -\boldsymbol{n}, J_0, J_3, \mu\right). \tag{S30}$$

The chiral symmetry is defined by the product of the particle-hole symmetry and the time-reversal symmetry,

$$\Pi = \Theta\Xi = i\tau_y, \tag{S31}$$

which transforms the Hamiltonian as

$$\Pi H_{3D} \left(\boldsymbol{k}, \boldsymbol{n}, J_0, J_3, \mu\right) \Pi^{-1} = -H_{3D} \left(\boldsymbol{k}, -\boldsymbol{n}, J_0, -J_3, -\mu\right). \tag{S32}$$

The chiral transformation is preserved when $\boldsymbol{n} = \boldsymbol{0}$, $J_0 = 0$ and $\mu = 0$.

We make the mirror symmetry along y direction

$$\sigma_y \mapsto -\sigma_y, \quad n_y \mapsto -n_y. \tag{S33}$$

The domain wall energy is the same for ϕ and $2\pi - \phi$ due to the mirror symmetry along the y direction as $\sigma_y \mapsto -\sigma_y$, $n_y \mapsto -n_y$. Therefore it is enough to show the results for $0 \leq \phi \leq \pi$.

2D Hamiltonian:

The 2D Hamiltonian for the surface state is given by

$$H_{2D} \left(\boldsymbol{k}, \boldsymbol{n}, \mu\right) = \hbar v \left(k_x \sigma_y - k_y \sigma_x\right) + n_x \sigma_x + n_y \sigma_y + n_z \sigma_z - \mu. \tag{S34}$$

Its complex conjugate is

$$H_{2D}^* \left(\boldsymbol{k}, \boldsymbol{n}, \mu\right) = \hbar v \left(-k_x \sigma_y - k_y \sigma_x\right) + n_x \sigma_x - n_y \sigma_y + n_z \sigma_z - \mu. \tag{S35}$$

The time-reversal operator is given by

$$\Theta = i\sigma_y K, \tag{S36}$$

which transforms the Hamiltonian as

$$\Theta H_{2D} \left(\boldsymbol{k}, \boldsymbol{n}, \mu\right) \Theta^{-1} = \sigma_y H_{2D}^* \left(\boldsymbol{k}\right) \sigma_y = \hbar v \left(-k_x \sigma_y + k_y \sigma_x\right) - n_x \sigma_x - n_y \sigma_y - n_z \sigma_z - \mu = H_{2D} \left(-\boldsymbol{k}, -\boldsymbol{n}, \mu\right). \tag{S37}$$

When $\boldsymbol{n} = \boldsymbol{0}$, the time-reversal transformation symmetry is preserved,

$$\Theta H_{2D} \left(\boldsymbol{k}, \boldsymbol{n} = \boldsymbol{0}, \mu\right) \Theta^{-1} = H_{2D} \left(-\boldsymbol{k}, \boldsymbol{n} = \boldsymbol{0}, \mu\right). \tag{S38}$$

On the other hand, when $\mu = 0$, there is a symmetry

$$\Theta H_{2D} \left(\boldsymbol{k}, \boldsymbol{n}, \mu = 0\right) \Theta^{-1} = -H_{2D} \left(\boldsymbol{k}, \boldsymbol{n}, \mu = 0\right) \tag{S39}$$

with the time-reversal symmetry operator Θ even when $\boldsymbol{n} \neq \boldsymbol{0}$.

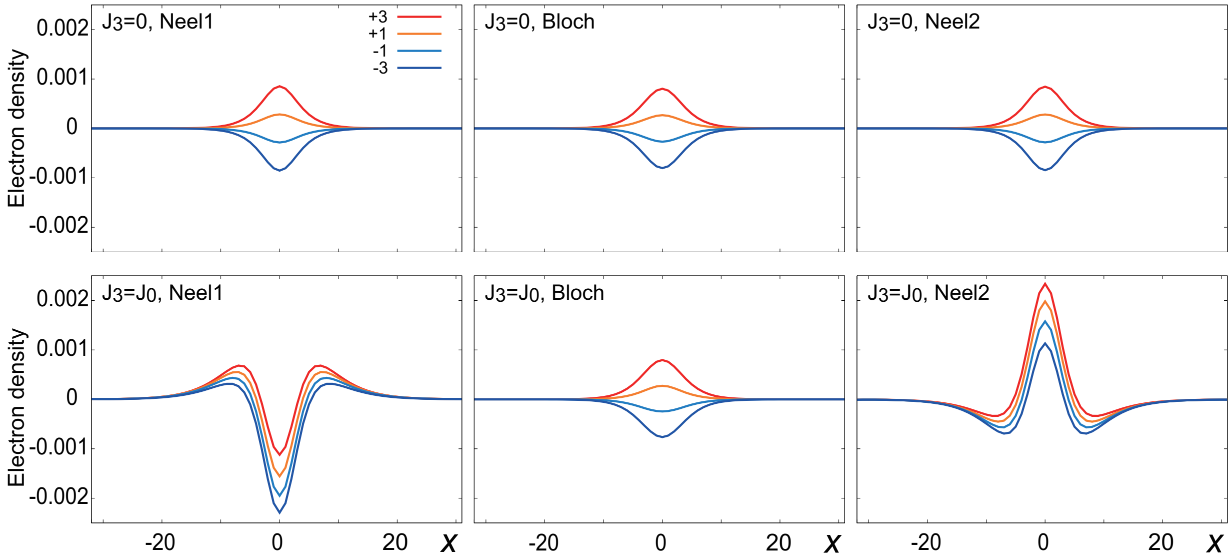

FIG. S5: Electron density distribution for various domain wall structures. The electron density distribution is almost the same between the case of $J_3 = 0$ and the Bloch domain wall with $J_3 = J_0$ because the $\mathrm{div}\boldsymbol{n}$ term is zero. There is a double peak structure for the $J_3 = J_0$ Neel wall due to the $\mathrm{div}\boldsymbol{n}$ term.

S6. Electron accumulation for the Neel wall and the Bloch wall

Figure S5 shows the electron density distribution for the cases of Neel and Bloch walls. There are two peak structures for $J_3 = J_0$ for the Neel domain wall, which come from $\mathrm{div}\boldsymbol{n}$ term in Eq. (22) in the main text.

S7. Charging energy

We evaluate the charging energy for three cases: (i) heavily doped case where the Fermi energy is in the 2D conduction band, (ii) half-filling case, and (iii) the case where the Fermi energy is deviated from the half-filling but in the 2D bang gap, and show that the charging energy can be neglected compared with the band energy.

We take the lattice constant $a \approx 10$ Å as the unit. Then, the unit of the Coulomb energy is

$$V = \frac{e^2}{a\varepsilon} = \frac{2a_B}{\varepsilon a}\frac{e^2}{2a_B} \approx 0.1\text{eV}, \tag{S40}$$

where, we have used $\varepsilon \approx 20$ for the TIs.

(i) Heavily doped case. For the heavily doped case, we can neglect the long-range Coulomb interaction and consider only the onsite repulsion because of the screening. According to numerical calculations, the electron distribution amplitudes is less than 10^{-2}, and the width is about 10. Therefore, the charging energy is

$$E_{\text{charge}} = V \times 2 \times 10 \times \left(10^{-2}\right)^2 = 2 \times 10^{-4}\text{eV}, \tag{S41}$$

where we have taken into account the two domain walls due to the periodic boundary condition. On the other hand, the order of the band energy is

$$E_{\text{band}} = 0.004 \times 0.3 = 1.2 \times 10^{-3}\text{eV}, \tag{S42}$$

where we have used $t \approx 3$eV Å for the TIs. E_{band} is about ten times larger than E_{charge}. Therefore, we can neglect the effect of the charging energy.

(ii) Half-filling case. We have to consider the long-range Coulomb interaction.

$$E_{\text{charge}} = V\int dxdx' \int_0^L dydy' \frac{\rho(x)\rho(x')}{\sqrt{(x-x')^2+(y-y')^2}} \tag{S43}$$
$$\approx 2LV\int dxdx' \left(\log\frac{2L}{|x-x'|}-1\right)\rho(x)\rho(x') \tag{S44}$$
$$\approx 2LV\rho_0^2\int_{-\xi/2}^{\xi/2} dxdx' \left(\log\frac{2L}{|x-x'|}-1\right) \tag{S45}$$
$$= 2LV\left(\rho_0\xi/2\right)^2\int_{-1}^{1} dxdx' \left(\log\frac{4L}{\xi}-\log|x-x'|-1\right) \tag{S46}$$
$$= 2LV\left(\rho_0\xi\right)^2\log\frac{4L}{\xi}+(\xi-\text{independent term})\,. \tag{S47}$$

On the other hand, the band energy is calculated as

$$E_{\text{band}} = \xi L\int_0^{J_\perp} d\varepsilon\varepsilon D\left(\varepsilon\right) = \frac{{J_\perp}^3}{6\pi v_{\text{F}}^2}L\xi \equiv \alpha L\xi, \tag{S48}$$

where we have used the density of states $D\left(\varepsilon\right)=\frac{\varepsilon^2}{2\pi v_{\text{F}}^2}$ for the Weyl Hamiltonian. Therefore, E_{band} is proportional to ξ as in the Fig. S2. If we use the parameter used in the calculation, we get $\alpha\approx 4\times10^{-4}$. In the Fig. S2, we obtain $\alpha_1 = 2\times10^{-2}$ for the Ising case and $\alpha_2 = 3\times10^{-3}$ for the Heisenberg case. In the evaluation, we use the numerical result.

We obtain the optimized ξ by minimizing $E_{\text{band}}+E_{\text{charge}}$.

$$\xi_{\text{opt}} = \frac{2V\left(\rho_0\xi\right)^2}{\alpha}. \tag{S49}$$

For the half-filling case, only the $\nabla\cdot\boldsymbol{n}$ term contributes the charging, and the amplitude is less than $1/L_y = 1/200$, and $\rho_0\xi\approx 1/200\times10 = 0.05$. Therefore, we get the optimized ξ as

$$\xi_{\text{opt}} = \frac{2\times2\times0.1\times\left(0.05\right)^2}{\alpha} = 0.3 \tag{S50}$$

for the Heisenberg case. Therefore, we conclude that the effect of the charging energy is negligible for the half-filling case.

(iii) The case where the Fermi energy is in the 2D gap. In this case, the Jackiw–Rebbi solutions accumulate. We can estimate $\rho_0\xi\approx N/L$ for the N electron doped system. On the other hand, the particle number is expressed as the function of the Fermi energy.

$$N = \xi L\int_0^{\mu} d\varepsilon D\left(\varepsilon\right) = \xi L\frac{\mu^2}{4\pi v_{\text{F}}^2}. \tag{S51}$$

Therefore,

$$\xi_{\text{opt}} \approx \frac{2V}{\alpha}\left(\xi^2\frac{\mu^2}{4\pi v_{\text{F}}^2}\right)^2. \tag{S52}$$

The Fermi energy which gives $\xi_{\text{opt}}\approx 1$ is $\mu\approx 0.3$eV. It is much larger than the surface gap (≈ 50 meV). Therefore, the charging energy is negligible for the arbitrary Fermi energy.

S8. Layer dependence of the domain wall energy

Figure S6 shows the domain wall energy E_{DW} for various depths of the magnetic layer. In the case of the second and middle layers, the energy differences are small because the amplitudes of surface states are small. The domain wall energy E_{DW} for the top layer with ϕ is identical to that of the bottom layer with $\pi-\phi$, while the domain wall energy of the middle layer is symmetric between ϕ and $\pi-\phi$, because of the mirror symmetry at the middle layer.

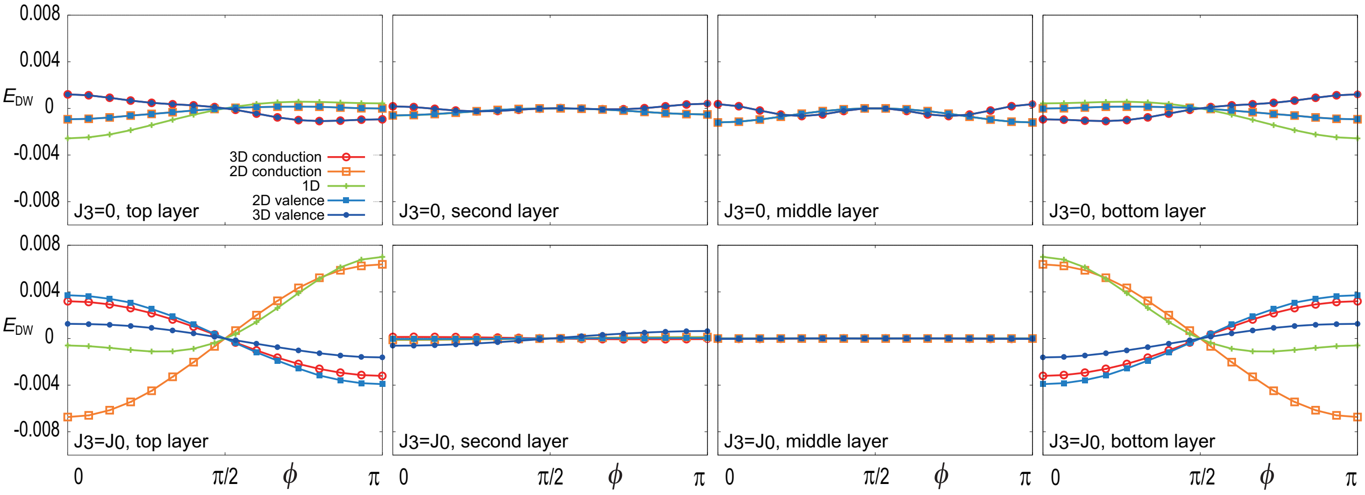

FIG. S6: Domain wall energy $E_{\rm DW}$ for various depths of the magnetic layer. The figures for the top layer and the bottom layer are symmetric with respect to $\phi \to \pi - \phi$, while the figure of the middle layer is symmetric around $\phi = \pi/2$ because of the mirror symmetry at the middle layer.
